# Extracts from *Acacia catechu* suppress HIV-1 replication by inhibiting the activities of the viral protease and Tat

**DOI:** 10.1186/1743-422X-10-309

**Published:** 2013-10-18

**Authors:** Nutan, Manoj Modi, Charlene S Dezzutti, Shweta Kulshreshtha, Ajay Kumar Singh Rawat, Sharad Kumar Srivastava, Swadesh Malhotra, Anjali Verma, Udaykumar Ranga, Satish Kumar Gupta

**Affiliations:** 1 Reproductive Cell Biology Laboratory, National Institute of Immunology, Aruna Asaf Ali Marg, New Delhi 110 067, India; 2 Magee-Womens Research Institute, University of Pittsburgh, Pittsburgh, PA 15213, USA; 3 National Botanical Research Institute, Rana Pratap Marg, Lucknow-226 001, Uttar Pradesh, India; 4 HIV-AIDS Lab, Molecular Biology and Genetics Unit, Jawaharlal Nehru Centre for Advanced Scientific Research, Jakkur, Bangalore 560 064, India

## Abstract

**Background:**

*Acacia catechu* (Mimosa family) stem bark extracts have been used traditionally as a dietary supplement as well as a folk medicine given its reported anti-inflammatory, immunomodulatory, hepatoprotective, antioxidant, anti-microbial and anti-tumor activities. The present study was undertaken to evaluate the anti-HIV-1 activity of the extracts from stem bark of *A. catechu*.

**Methods:**

The aqueous and 50% ethanolic extracts of *A. catechu* stem bark were prepared and 50% ethanolic extract was further fractioned by successively partitioning with petroleum ether, chloroform and n-butanol. All the extracts and fractions were evaluated for cytotoxicity and anti-HIV-1 activity using different *in vitro* assays. The active n-butanol fraction was evaluated for its inhibition against HIV-1 reverse transcriptase, integrase, protease, pro-viral genome integration and viral Tat protein mediated transactivation. The effect of n-butanol fraction on the induction of pro-inflammatory cytokines secretion in Vk2/E6E7 cells and transepithelial resistance in Caco-2 and HEC-1A cells was investigated.

**Results:**

The aqueous and 50% ethanolic extracts of *A. catechu* showed IC_50_ values of 1.8 ± 0.18 μg/ml and 3.6 ± 0.31 μg/ml, respectively in cell-free virus based assay using TZM-bl cells and HIV-1_NL4.3_ (X-4 tropic). In the above assay, n-butanol fraction exhibited anti-HIV-1 activity with an IC_50_ of 1.7 ± 0.12 μg/ml. The n-butanol fraction showed a dose-dependent inhibition against HIV-1_NL4.3_ infection of the peripheral blood lymphocytes and against HIV-1_BaL_(R-5-tropic) as well as two different primary viral isolates of HIV-1 infection of TZM-bl cells. The n-butanol fraction demonstrates a potent inhibitory activity against the viral protease (IC_50_ = 12.9 μg/ml), but not reverse transcriptase or integrase. Further, in Alu-PCR no effect on viral integration was observed. The n-butanol fraction interfered with the Tat-mediated Long Terminal Repeat transactivation in TZM-bl cells, mRNA quantitation (qRT-PCR) and electrophoretic mobility shift assay (EMSA). The n-butanol fraction did not cause an enhanced secretion of pro-inflammatory cytokines in Vk2/E6E7 cells. Additionally, no adverse effects were observed to the monolayer formed by the Caco-2 and HEC-1A epithelial cells.

**Conclusions:**

The results presented here show a potential anti-HIV-1 activity of *A. catechu* mediated by the inhibition of the functions of the viral protein and Tat.

## Background

Highly active antiretroviral therapy (HAART) has led to a dramatic increase in the longevity and the quality of life for people infected with HIV-1 [1], but due to the emergence of drug resistant virus [2], there is a continuous need to develop new anti-HIV-1 agents with novel targets and mechanisms of action. Topical application of micobicides not only prevents the viral infection at the portal of entry but also may empower women with decision making. Since natural products have an enormous structural diversity and provide a large reservoir for new therapeutic/preventive regimens, exploring them for the targets against HIV-1 infection is a promising option [3-6].

The early events in HIV-1 life-cycle comprise of the viral attachment to the host cell surface followed by the conversion of the viral RNA genome into proviral DNA by the virally-encoded enzyme, reverse transcriptase (RT), and its integration in the host genome by the virally-encoded enzyme integrase [7,8]. The provirus integrated in the host genome may remain in a quiescent state in the resting lymphocytes until basal transcription produces a threshold level of the viral trans-activator protein, Tat. As Tat accumulates above the threshold, it leads to the transition from latent state of HIV-1 to active replication in lymphocytes when the protein interacts with the Tat-responsive element (TAR) located in the long terminal repeat (LTR) promoter in the viral DNA [9-11]. Extracellular Tat has also been implicated in acquired immunodeficiency syndrome (AIDS) and AIDS-associated pathologies [12]. The late events of the viral life cycle include the processes of HIV-1 mRNA synthesis, protein expression and virus maturation. The progeny viruses expressed from the activated viral gene expression are assembled on and budded through the host cell membrane after being processed by the viral encoded enzyme protease [7]. Compounds that block activation or suppression of the viral gene expression have a therapeutic potential for extension of latency or inhibition of persistent progressive infection. Discovering drugs that interfere with the functionality of the crucial enzymes of HIV-1 that play a critical role in viral pathogenesis i.e. RT, integrase and protease are important targets to be considered against HIV-1 infection.

*Acacia catechu*, commonly known as catechu, cachou and black cutch is an important medicinal plant, especially prevalent in Asia. It is an extensively studied plant. A wide spectrum of compounds that have been isolated and characterized from *A. catechu* include 4-hydroxybenzoic acid, kaempferol, quercetin, 3,4',7-trihydroxyl-3′,5-dimethoxyflavone, catechin, rutin, isorhamnetin, epicatechin, afzelechin, epiafzelechin, mesquitol, ophioglonin, aromadendrin and phenol [13]. Catechins, rutin and isorhamnetin exhibit antioxidant property by scavenging free-radicals [14]. The flavonoid rich extract of *A. catechu* mainly comprised of catechins demonstrated anti-inflammatory activity by reducing the production of pro-inflammatory eicosanoids [15] as well as immunomodulatory property with a significant effect on cell mediated and humoral immunity against foreign antigens [16,17]. The methanolic extract of this plant possesses antimicrobial activity against different species of pathogenic and non-pathogenic microorganisms [18] as well as DNA protective activities [19]. Anti-fertility activity of a traditional contraceptive pill comprising *A. catechu* has also been reported [20].

In context of the ethnopharmacological importance of *A. catechu*, we screened its aqueous and ethanolic extracts for anti-HIV-1 activity. Further, the active n-butanol fraction was studied for its ability to block HIV-1 infection and the possible mechanisms of action.

## Results

### Cytotoxicity and anti-HIV-1 activity of extracts, fractions and compounds from *A. catechu*

**Figure 1:**
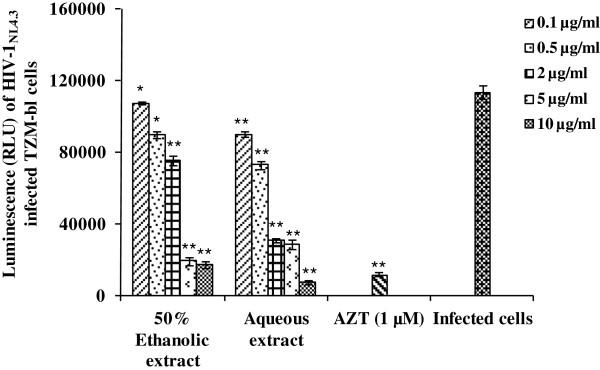
**Anti-HIV-1 activity of *Acacia catechu *extracts.** TZM-bl cells were infected with HIV-1_NL4.3_ (MOI, 0.05) pretreated for 1 h with extracts prepared from *A. catechu* and incubated for 48 h in the presence or absence of extracts as described in *Materials and Methods*. Anti-HIV-1 activity at varying concentrations of the 50% ethanolic, aqueous stem bark extracts and AZT (1 μM), used as a positive reference control, has been shown. Y-axis represents the percent inhibition of HIV-1 infection. Values are expressed as mean ± SE of 3 different experiments performed in duplicates. *p < 0.05; **p < 0.001 (as compared to infected control group without any treatment).

Our interest was to systematically evaluate aqueous and 50% ethanolic extracts prepared from stem bark of *A. catechu* for activity against HIV-1 after their initial characterization. Multiple peaks were observed in the reverse phase HPLC profiles of both aqueous as well as 50% ethanolic extracts of *A. catechu* stem bark, at an isocratic gradient (Additional file 1: Figure S1). The 50% ethanolic extract appeared to be more complex as compared to that of aqueous extract. The initial screening for anti-HIV-1 activity of the *A. catechu* extracts was performed using reporter-gene based TZM-bl cells. Extracts were first screened for their impact on cellular viability. CC_50_ is the concentration of the extracts that reduced the cell viability by 50%. The 50% ethanolic extract showed CC_50_ at 371.0 ± 11.7 μg/ml whereas the aqueous extract was non-toxic up to 400 μg/ml for TZM-bl cells (Table 1). A dose-dependent inhibition in HIV-1_NL4.3_ infection by the aqueous as well as 50% ethanolic extracts was observed (Figure 1). The 50% inhibitory concentrations (IC_50_) of the 50% ethanolic and aqueous extracts were 3.6 ± 0.31 and 1.8 ± 0.18 μg/ml, respectively (Table 1).

**Table 1:** **Cytotoxicity and anti-HIV-1 activity of the extracts/n-butanol fraction of stem bark of *A. catechu *** **using HIV**_**NL4.3 **_**in TZM-bl cells based assay**

**Treatment group**	** TZM-bl cells (μg/ml)**		
	*CC_**50**_	*IC_**50**_	**TI**
50% Ethanolic extract	371.0 ±11.7	3.6 ± 0.31	103.0 ± 6.3
Aqueous extract	>400**	1.8 ± 0.18	>200
n-Butanol fraction	>400**	1.7 ± 0.12	>200

*CC_*50*_: *The cytotoxic concentration of the extracts/n-butanol fraction that caused the reduction of viable cells by 50%. All data presented are averages of three independent experiments done in duplicates (mean ± standard error).*

*IC_*50*_: *The concentration of the extracts/n-butanol fraction that resulted in 50% inhibition in HIV-1 infection. All data presented are averages of three independent experiments done in duplicates (mean ± standard error).*

**CC_*50*_*was not obtained as there was no toxicity up to the maximum concentration tested.*

*TI: Therapeutic index is CC*_*50*_*/IC*_*50.*_

**Figure 2:**
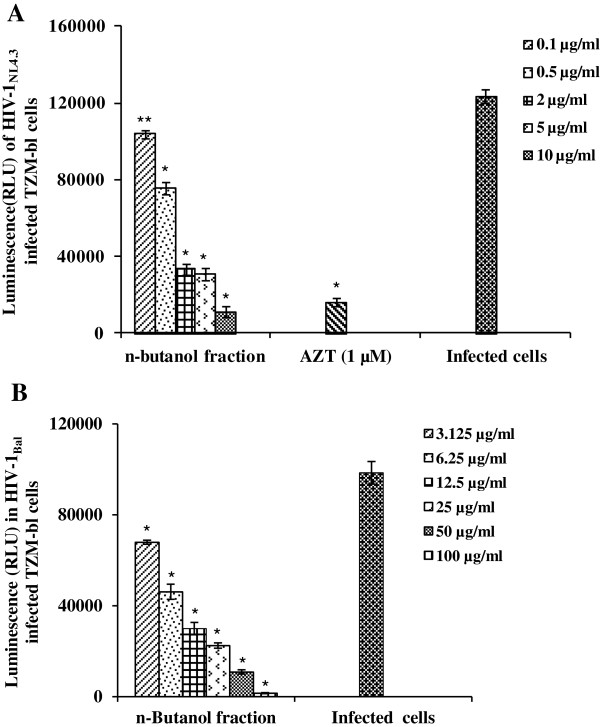
**Anti-HIV-1 activity of n-butanol fraction of *Acacia catechu*. A)** Anti-HIV-1_NL4.3_ activity of n-butanol soluble fraction at different concentrations in TZM-bl cells. **B)** Inhibition against HIV-1_BaL_ infection using TZM-bl cells. Y-axis represents the luciferase expression in terms of relative luminescence unit (RLU) in HIV-1 infected and treated TZM-bl cells whereas X-axis represents different concentrations of the butanol fraction. Values are expressed as mean ± SE of 3 different experiments performed in duplicates. *p < 0.05; **p < 0.001 (as compared to infected control group without any treatment).

**Figure 3:**
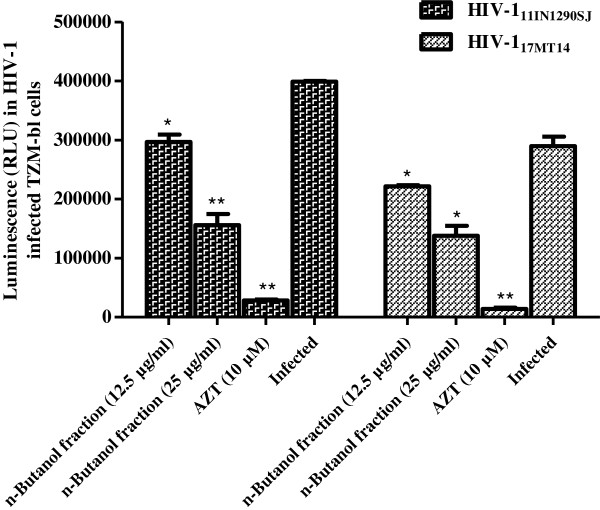
**Anti-HIV-1 activity of n-butanol fraction of *Acacia catechu *against CCR5-tropic clinical isolates.** Anti-HIV-1 activity of n-butanol soluble fraction of *A. catechu* tested at two different concentrations/AZT (10 μM) against clinical isolates namely HIV-1_11IN1290SJ_ and HIV-1_17MT14_, using TZM-bl cells. Y-axis represents the luciferase expression as RLU in HIV-1 infected and treated TZM-bl cells. Values are expressed as mean ± SE of 3 different experiments performed in duplicate. *p < 0.05; **p < 0.01 (as compared to infected control group without any treatment).

**Figure 4:**
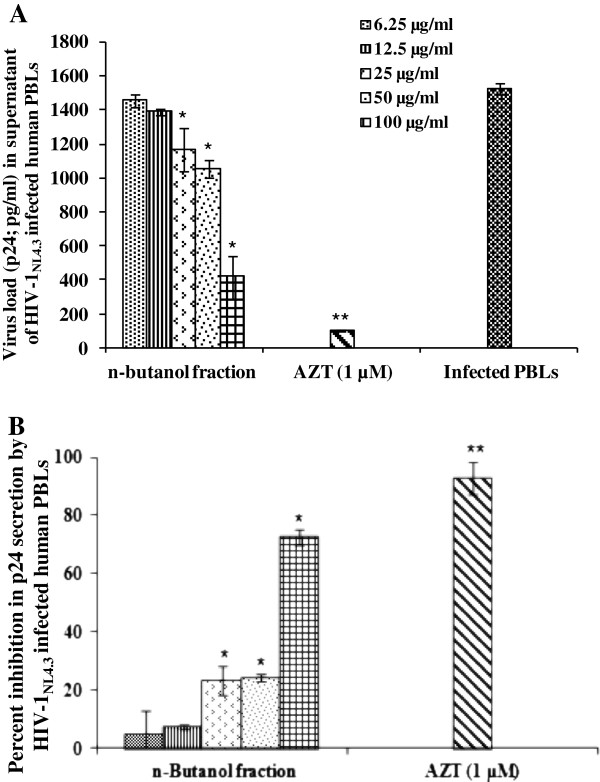
**Anti-HIV-1 activity of n-butanol fraction of *Acacia catechu *using human peripheral blood lymphocytes (PBLs). A)** Anti-viral effect of the n-butanol fraction using peripheral blood lymphocytes (PBLs) against HIV-1_NL4.3_. PHA-P activated PBLs were infected with virus and were treated with different concentration of n-butanol fraction from *A. catechu*. p24 was estimated on 5^th^ day in the culture supernatant. Significance (p-value) was calculated by comparison with amount of p24 in the infected control group. **B)** The amount of p24 secreted by infected PBLs in term of percent inhibition presented on the Y-axis. Values are expressed as mean ± SE of 3 different experiments. *p < 0.05; **p < 0.001 (as compared to infected control group without any treatment).

Of the three fractions, petroleum ether, chloroform and n-butanol soluble fractions prepared from the 50% ethanolic extract of stem bark, HPLC profiles suggest the chloroform fraction to be the most complex, with maximum number of peaks (Additional file 2: Figure S2). However, the n-butanol fraction demonstrated better cell viability and anti-HIV-1 activity. There was no toxicity observed by the n-butanol fraction up to a maximum concentration of 400 μg/ml on TZM-bl cells (Table 1). A dose-dependent inhibition was observed with an IC_50_ value of 1.7 ± 0.12 μg/ml using TZM-bl cell based assay (Figure 2A; Table 1). Further, the n-butanol fraction exhibited a marked decrease in HIV-1_BaL_ (R-5 tropic) infection, at non-cytotoxic concentrations in TZM-bl cells (Figure 2B). The activity of n-butanol fraction was also evaluated against two primary isolates of CCR5-tropic HIV-1 (11IN1290SJ & 17MT14), where potent inhibition was also observed against both the isolates (Figure 3). Further, to confirm the efficacy of the n-butanol fraction of *A. catechu* in primary cells, PHA-P activated human peripheral blood lymphocytes (PBLs) based assay was used. The n-butanol fraction (100 μg/ml) reduced the p24 concentration to 417 pg/ml as compared to 1525 pg/ml in control (infected PBLs without any treatment; Figure 4A) i.e. 73% inhibition in HIV-1 infection (Figure 4B). Anti-HIV-1 activity of the n-butanol fraction in human PBLs was not due to non-specific cytotoxicity as more than 80% cells were viable when fraction was used at 100 μg/ml (Additional file 3: Figure S3). In an initial attempt, to isolate and characterize pure compounds responsible for anti-HIV-1 activity, five different compounds were purified and screened for their anti-HIV-1 activity using TZM-bl cells in cell-free virus based assay against HIV-1_NL4.3_ (Table 2). Among these compounds, only catechins exhibited a potent anti-HIV-1 activity with CC_50_ and IC_50_ values of 950 μg/ml and 0.6 μg/ml, respectively. Investigations for further identification of other anti-HIV-1 active compounds from the 50% ethanolic extract of stem bark are under progress.

**Table 2:** **Different compounds isolated from 50% ethanolic stem bark extract of *Acacia catechu***

**Compounds**	**Anti-HIV-1**	**Physical**	**Chemical Family**
	**activity**	**aspect**	
Catechins	CC_50_ = 950 μg/ml	White powder	Flavonoids; (+)-catechin; & (−)- epicatechin (cis)
	IC_50_ = 0.60 μg/ml		
Kaempferol	No	Yellow crystalline powder	Flavonoid
Rutin	No	Yellow powder	Flavonoid glycoside
Ferulic acid	No	Yellow-white crystalline powder	Polyphenol
Caeffic acid	No	Yellow powder	Polyphenol

### Probable mechanism of action of the active fraction of *A. catechu*

**Figure 5:**
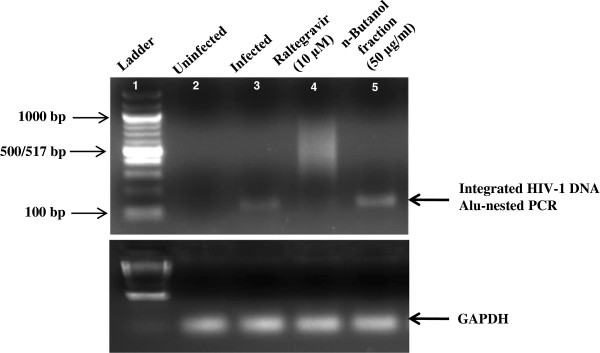
**Effect of n-butanol fraction prepared from *A. catechu *on integration of HIV-1 genome in host cells.** TZM-bl cells were infected with HIV-1_NL4.3_ (MOI, 0.05) pretreated for 1 h with n-butanol fraction prepared from *A. catechu* (50 μg/ml) and Raltegravir (10 μM), used as a positive reference control and incubated for 48 h in the presence or absence of extract/Raltegravir as described in *Materials and Methods*. After 48 h, cells were trypsinized and washed. Genomic DNA was isolated from HIV-uninfected/infected cells. DNA (200 ng) was used as a template and viral integration was monitored by Alu-LTR-PCR with Alu-gag primers and GAPDH was used as internal control.

Further studies were performed to delineate the mechanisms by which the extracts/n-butanol fraction prepared from *A. catechu* inhibited HIV-1 infection. The inhibition by active n-butanol fraction against HIV-1 entry and inhibition of the HIV-1 enzymes; RT, integrase and protease were tested. The active fraction did not show any inhibition of HIV-1_NL4.3_ entering into the host cells (TZM-bl) even at a dosage of 50 μg/ml, when compared with the known entry inhibitor Bicyclam, which was 95% effective even at 0.4 μg/ml (data not shown). In cell-based fusion assay that mimic the gp120-CD4-mediated fusion [21], pre-treatment of HL2/3 cells (expressing HIV-1 Env on their surface and Tat protein in cytoplasm) with n-butanol fraction (10 μg/ml) did not lead to any significant inhibition in fusion with TZM-bl cells as determined by estimation of luciferase expression (Additional file 4: Figure S4a). We next determined whether the n-butanol fraction directly inhibited HIV-1 RT activity. Nevirapine (1 μM), a potent RT inhibitor, inhibited HIV-1 RT activity by 72%; however, n-butanol fraction did not show any effect on the RT activity (Additional file 4: Figure S4b). These results imply that inhibition of HIV-1 replication by the n-butanol fraction was not due to its effects on the RT activity but on other steps of HIV-1 life cycle. To evaluate the effect of n-butanol fraction on HIV-1 integrase, sodium azide (0.1%) was used as a reference control that exhibited >90% inhibition against HIV-1 integrase activity whereas the n-butanol fraction (50 μg/ml) did not show any significant inhibition of integrase activity (Additional file 4: Figure S4c). The lack of effect on viral integration was confirmed by treating TZM-bl cells infected with HIV-1_NL4.3_, with either the n-butanol fraction or vehicle control. Viral integration was monitored by Alu-LTR PCR (Figure 5). As compared to the positive reference control Raltegravir (10 μM; a HIV-1 integrase inhibitor), there was no effect of the n-butanol fraction on the virus integration.

**Figure 6:**
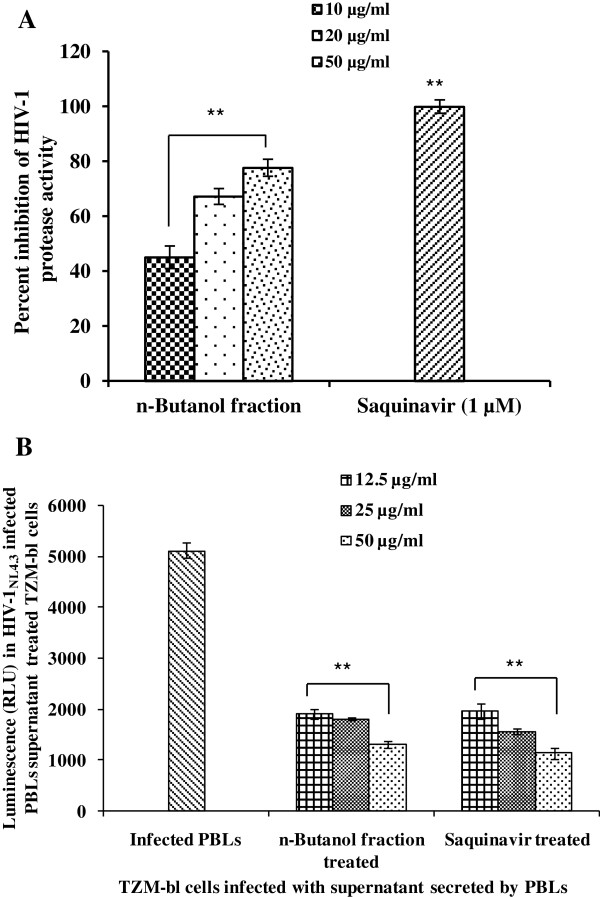
**Effect of n-butanol fraction of *A. catechu *on HIV-1 protease activity and maturation of virion particles. A)** Dose dependent inhibition in HIV-1 protease activity by the n-butanol fraction as compared with Saquinavir used as a reference control in a commercial ELISA kit. **p < 0.01 (as compared to enzyme only control). **B)** Comparison of the infectivity of TZM-bl cells by the virions released from the infected and n-butanol fraction/Saquinavir (a protease inhibitor) treated PBLs. The culture supernatant collected on 5^th^ day was used to infect TZM-bl cells for 48 hours and relative luminescence unit (RLU) was calculated by lysing the cells as described in *Materials and Methods*. Y-axis represents the luciferase expression as RLU. Values are expressed as mean ± SE of 2 different experiments performed in duplicate. **p < 0.01 (as compared to cells treated with soup from infected control group without any treatment).

Finally, the effect of the n-butanol fraction was evaluated on HIV-1 protease activity. The n-butanol fraction showed a significant reduction in HIV-1 protease activity with an IC_50_ value of 12.9 μg/ml (Figure 6A). The standard HIV-1 protease inhibitor drug, Saquinavir (1 μM) was used as a reference control that showed 100% inhibition. To validate the effect of active fraction on HIV-1 protease activity, release of mature viral particles by HIV-1 infected and extract treated PBLs was investigated. The culture supernatant was used to infect TZM-bl cells to quantify the infectivity of the released virions and hence correlate it with the HIV-1 protease activity. As compared with the culture supernatant obtained from untreated PBLs there was a decrease in the expression of luciferase when culture supernatant from PBL treated with n-butanol fraction was used (Figure 6B). Similar results were observed with the culture supernatant from HIV-1 infected PBL but treated with Saquinavir (Figure 6B).

**Figure 7:**
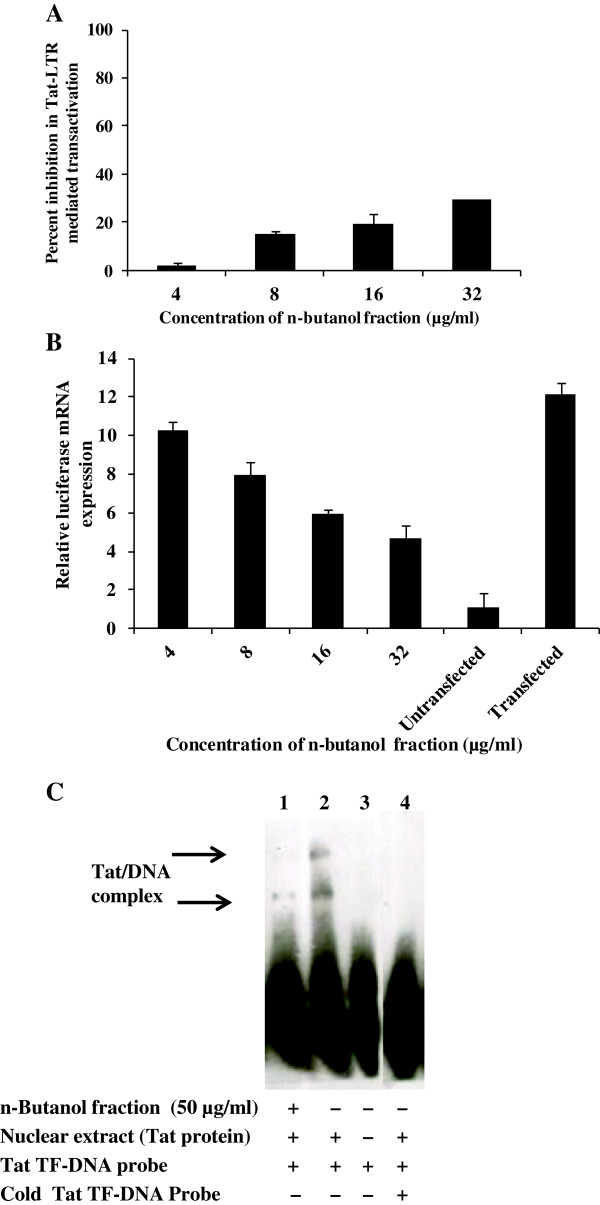
**Study of mechanism of action of the n-butanol fraction of *Acacia catechu*. A)** Inhibitory activity of n-butanol fraction against Tat-mediated HIV-1 genes transactivation by pTat transfection in TZM-bl cells. Values are expressed as average of 2 different experiments performed in duplicates. **B)** The effect of n-butanol fraction on luciferase gene expression at mRNA level. The n-butanol fraction prepared from 50% ethanolic extract of stem bark of *A. catechu* was added to TZM-bl cells post-transfection with pTat. Reduction in the expression of luciferase gene was validated by qRT-PCR. qRT-PCR data is expressed as fold change in expression as compared to control. Values are expressed as mean ± SE of 2 different experiments performed in duplicate. **C)** The inhibitory activity of n-butanol fraction using Tat based Electrophoretic Mobility Shift Assay (EMSA). Tat protein was incubated with DNA probe in presence or absence of the active n-butanol fraction of the plant. Samples were run in 6% non-denaturing polyacrylamide gel. Lane 1 is with Tat transcription factor (TF) DNA probe + Tat protein (nuclear extract prepared from transfected TZM-bl cells) + n-butanol fraction (50 μg/ml); Lane 2, TF DNA probe + Tat protein; Lane 3, TF DNA probe only; Lane 4 - TF DNA probe + Cold TF DNA probe + Tat protein.

To evaluate the inhibitory effect of extract/fraction on Tat mediated transactivation of HIV-1 gene expression, TZM-bl cells were transfected with pcDNA-Tat expression vector. In TZM-bl cells, luciferase reporter gene is under the control of LTR promoter and hence Tat-LTR mediated transactivation was determined by luciferase expression. The n-butanol fraction prepared from *A. catechu* stem bark showed a modest dose-dependent inhibition in luciferase expression (Figure 7A). Simultaneously, to see the reduction at mRNA level of luciferase gene (expression is under the control of Tat-LTR mediated activation), as a result of treatment of pcDNA-Tat transfected TZM-bl cells with n-butanol fraction, real-time PCR was performed as described in *Materials and Methods*. As expected, there was a dose dependent reduction in the level of luciferase mRNA in n-butanol fraction treated transfected cells when compared to untreated transfected cells (Figure 7B). Further in the Tat-EMSA, no bands were observed in Lane 1 (in presence of 50 μg/ml of n-butanol fraction) as compared to Lane 2 where bands appeared as a result of nuclear Tat protein interaction with DNA probe. These results demonstrated that the phytochemicals present in the stem bark n-butanol fraction interferes with the Tat-LTR interaction mediated transactivation of viral genes in addition to HIV-1 protease activity (Figure 7C).

### Preclinical safety study of n-butanol fraction of *A. catechu*

As an attempt to evaluate the toxic effect of extracts/fraction on vaginal keratinocytes Vk2/E6E7, MTT assay was used. No cytotoxicity was observed up to 100 μg/ml, by the extracts and the n-butanol fraction prepared from *A. catechu* (data not shown). Further to assess the adverse effect of these extracts/fraction on pro-inflammatory cytokine secretion, compared to the control, both the plant extracts as well as n-butanol fraction at 100 μg/ml did not lead to any increase in the secretion of IL-8. However, a substantial decrease in its secretion was observed by 50% ethanolic extract and n-butanol fraction of *A. catechu* (Table 3)*.* A decreased secretion of IL-6 was also observed in case of cells treated with 50% ethanolic stem bark extract (34.8 pg/ml) and its n-butanol fraction (8.8 pg/ml) as compared to control value (118.0 pg/ml; Table 3). However, no change in the secretion of IL-1β and TNF by vaginal keratinocytes was observed with any of the treatment group at 100 μg/ml (Table 3).

**Table 3:** **Pro-inflammatory cytokines secretion by vaginal keratinocytes after treatment for 24 h with extracts/n-butanol fraction prepared from stem bark of *A. catechu***

Treatment with plant extract/fraction*	**Level of cytokines (pg/ml)**			
	**IL-8**	**IL-1β**	**IL-6**	**TNF**
**Control**	**282** ± **5.3**	**2.2 ± 0.2**	**118 ± 9.1**	**1.1 ± 0.6**
Aqueous extract	248 ± 11.1	3.4 ± 0.2	115.0 ± 8.4	1.7 ± 0.1
50% Ethanolic extract	40.4 ± 3.9**	4.0 ± 0.0	34.8 ± 1.3#	1.7 ± 0.3
n-Butanol fraction	2.7 ± 0.6**	3.4 ± 0.3	8.8 ± 0.6#	1.8 ± 0.5

*Various plant extracts/n-butanol fraction were used at 100 μg/ml.

**p < 0.001 as compared to IL-8 concentration in the control group.

#*p < 0.001 as compared to IL-6 concentration in the control group.*

**Figure 8:**
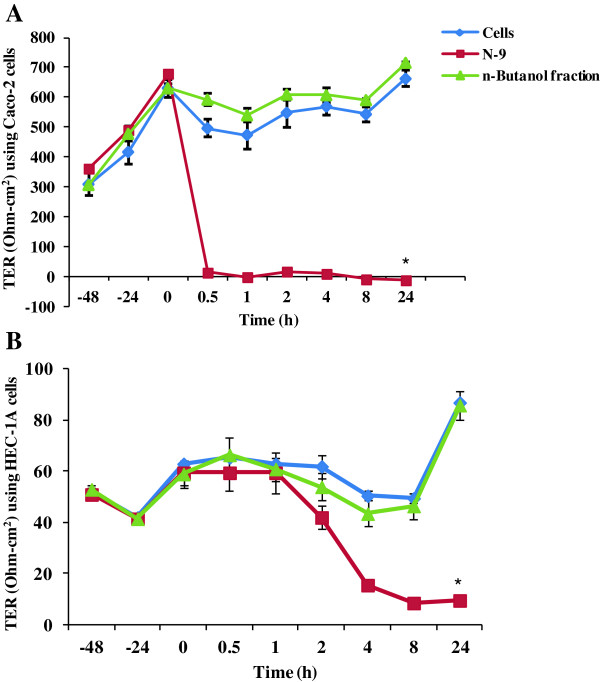
**Effect of n-butanol fraction of *Acacia catechu *on transepithelial resistance on monolayer of epithelial cells. A)** The effect of n-butanol fraction on Caco-2 epithelial cells monolayer integrity. Plant n-butanol fraction/Nonoxynol-9 (N-9) was added to the apical chamber at t = 0 and transepithelial resistance (TER) readings were measured at 0.5, 1, 2, 4, 8 and 24 h. As toxicity control, N-9 gel (1:50 dilution ~ 0.6 mg/ml) was added to the indicated well. **B)** The effect of the n-butanol fraction on TER of HEC-1A cells. Values are expressed as mean ± SE of 2 different experiments performed in duplicates.

Measurement of transepithelial resistance (TER) has been done on the tight junctions formed by epithelial cells such as Caco-2, HEC-1A and MDCK-1 [22]. Nonoxynol-9 (N-9) originally developed as a spermicidal, with activity against sexually transmitted pathogens including HIV-1 led to epithelial disruption as well as toxicity to the rectal mucosa as reported by different studies, and hence was included as a positive control in this work [23]. The n-butanol fraction of *A. catechu* was non-toxic on both Caco-2 and HEC-1A cells when tested up to 100 μg/ml (data not shown). After application of the n-butanol fraction to the apical monolayer of Caco-2 cells (Figure 8A) and HEC-1A cells (Figure 8B), there was no measurable effect on TER as shown by the consistency of the treated monolayer as compared to the untreated control.

## Discussion

*A. catechu* is commonly known as *Mimosa catechu*, whose phytoconstituents possess several pharmacological properties [15,18,24,25]. *A. catechu*, a medicinally and economically important plant, may serve as novel source of anti-HIV-1 active compounds. Although, anti-HIV-1 activity has been reported previously from different species of the genus Acacia e.g. *Acacia confusa*[26], no such activity has been shown from *A. catechu*. Our interest was to systematically evaluate aqueous and alcohol extracts of *A. catechu* for activity against HIV-1. Both the extracts exhibited a dose-dependent inhibition of HIV-1 infection at concentrations ranging from 0.1-10 μg/ml against HIV-1_NL4.3_. Therapeutic indices (TI) of the 50% ethanolic and aqueous extracts prepared from stem bark were calculated as the ratio of CC_50_ to IC_50_. A TI of >100 by these extracts suggest the plant as a candidate for further evaluation (Table 1). Further, 50% ethanolic extract was partitioned to yield petroleum ether, chloroform and n-butanol soluble fractions. Out of the three fractions, the n-butanol fraction demonstrated better anti-HIV-1 potential. Further, the n-butanol fraction exhibited a marked decrease in HIV-1 infection by HIV-1_BaL_ (R-5 tropic) as well as displayed a potent inhibition of infection of primary isolates of CCR5-tropic HIV-1 at non-cytotoxic concentrations. In an attempt to identify the active compounds from stem bark of *A. catechu* responsible for anti-HIV-1 activity, a total of 6 compounds from 50% ethanolic extract, were isolated and characterized using different chromatographic and spectroscopic studies (Table 2). Different compounds like catechins, kaempferol, rutin, ferulic acid and caeffic acid have been isolated and characterized from *A. catechu*, among which catechins have shown a very potent anti-HIV-1 activity in its preliminary studies. Other compounds, however failed to inhibit HIV-1 infection in cell-free virus based assay (data not shown). In previous studies, catechins, epicatechin, epicatechin-3-*O*-gallate and epigallocatechin-3-*O*-gallate were reported as the predominant compounds present in *A. catechu*[27]. In different investigations by several other groups, catechins isolated from tea leaves have been reported to inhibit HIV-1 infection [28,29].

In an attempt, to explore the mode of inhibition of HIV-1 infection by the active fraction of *A. catechu*, different *in vitro* assays were performed. The n-butanol fraction failed to inhibit the viral entry to the host cells. Due to the essential role of HIV-1 RT in synthesizing the double-stranded proviral DNA from single-stranded HIV-1 RNA genome, it is a major target among the current anti-HIV-1 therapies. The n-butanol fraction did not exhibit inhibitory activity against RT enzyme. Further, the activity of the n-butanol fraction was assessed against HIV-1 integrase, an enzyme that leads to the integration of the reverse transcribed viral DNA into the genome of the cell and hence plays an essential role in HIV-1 replication. The n-butanol fraction showed no adverse effect on this enzyme activity. Suppression of HIV-1 infection by n-butanol fraction was not at the pre-integration or integration steps, which was confirmed by Alu-LTR PCR where the active extract did not inhibit integration of viral genome into host DNA. Hence, the inhibitory effect of n-butanol fraction may be at the post-integration stage.

HIV-1 protease, an aspartyl protease, is another crucial enzyme essential for the life cycle of HIV-1, as it cleaves newly synthesized poly-proteins to create the mature protein components of an infectious HIV-1 virion. Protease inhibitors bind to the active site of the viral protease enzyme, preventing the processing of viral particles into mature and functional form. Interestingly, the n-butanol fraction of *A. catechu* showed a potent inhibition of HIV-1 protease activity. This finding was further supported by the observations that there was a decrease in the release of mature viral particles by infected PBLs after treatment with the n-butanol fraction as shown by reduction of greater than 50% infectious virus in the TZM-bl assay. Catechins containing galloyl moieties from different plant sources have been reported to target several key proteins of HIV-1. Hence, they may inhibit sexual transmission of HIV-1 by interfering with HIV-1 protease activities [30].

Viral Tat protein plays a pivotal role in both the HIV-1 replication cycle and the pathogenesis of HIV-1 infection [31-33]. HIV-1 Tat protein transactivate HIV-1 LTR promoter by binding to the nascent RNA stem-loop structure known as the transactivator response region (TAR). It has also been shown to bind to NFκB enhancer sequence [33]. Hence, inhibiting Tat-mediated functions could be another critical step of intervention by *A. catechu*. Transfection of TZM-bl cells with pTat expression vector wherein luciferase expression is under the control Tat-LTR mediated activation, followed by n-butanol fraction treatment resulted in dose-dependent suppression of luciferase expression. This observation was further corroborated by the expression level of luciferase mRNA using qRT-PCR. The inhibition of Tat and LTR interaction was also confirmed by the Tat-EMSA experiment. Several plant-derived compounds such as quinolines as well as stilbene- and purine-derivatives have been reported before to illustrate anti-HIV-1 activity by inhibiting Tat-LTR interactions [31]. Our unpredicted finding that *A. catechu* stem bark has a dual inhibitory mechanism demonstrates that herbal drugs can be developed that simultaneously inhibits HIV-1 protease as well as Tat-LTR mediated HIV-1 transcription.

The effect of these extracts/n-butanol fractions on vaginal keratinocytes viability is highly relevant, if the active fraction is considered as a topical agent that may be applied for prevention of HIV-1 transmission (microbicides), as vaginal epithelial cells form part of the physical barrier that may impede the passage of cell-free or cell-associated HIV-1 into sub-epithelial tissues. An effective vaginal microbicide should also obviate pro-inflammatory responses that facilitate transepithelial viral penetration and its replication. Pro-inflammatory cytokines such as IL-1β, IL-6, IL-8 and TNF stimulate viral replication in latently infected cells and hence may constitute a considerable inducible HIV-1 reservoir in the genital secretions [34]. Therefore, it was important to rule out induction of inflammatory cytokines secretion after application of the plant extracts/n-butanol fraction on vaginal keratinocytes. There was no cytotoxicity observed by all the extracts/fraction up to 100 μg/ml and none of them led to a significantly enhanced secretion of pro-inflammatory cytokines as an effect to VK2/E6E7 cells treatment. Since both IL-6 and IL-8 are associated with an increased HIV-1 replication, this could be an added advantage of the active n-butanol fraction of *A. catechu* as an anti-HIV-1 microbicide [35,36]. The decrease in secretion of pro-inflammatory cytokines, IL-6 as well as IL-8, may be due to the catechins that are present in abundance in the stem bark of *A. catechu* and have been reported previously for their anti-inflammatory properties [15]. Preclinical safety investigations should also comprise evaluation of interference with epithelial integrity. Maintenance of an intact as well as polarized monolayer in presence of a potential microbicide candidate is an important factor to be considered as any damage in epithelial layer may allow infectious virus to reach the host target cells [22,23]. Observed transient decrease in TER of CaCo-2 cells in presence of n-butanol fraction, may be due to the differential sensitivity of CaCo-2 cells *versus* HEC-1A cells as shown previously with tenofovir gel [37]. With no deleterious effect on the epithelial barrier layer, the extracts as well as n-butanol fraction from *A. catechu* appears to be safe and suggests their utility as microbicide candidate.

## Materials and methods

### Collection of plant material

*A. catechu* plant material was collected three times from Balrampur, Uttar Pradesh, India. The voucher specimen (accession number NBRH-04) has been submitted to Herbarium of National Botanical Research Institute, Lucknow, India.

### Extraction and solvent fractionation of plant material

Air and shed dried stem bark from *A. catechu* was grinded and strained through 30 mesh (0.5 mm). The finely grinded stem bark (100 gm) was treated with 500 ml MilliQ water at 60-75°C for 6–8 h. This process was repeated three more times. The hot water extract was filtered through Whatman filter paper number 1. The combined filtrate (1300 ml) was distilled at 45-50°C under vacuum to concentrate the extract to 300 ml. The extract was then lyophilized at −20 to −40°C to afford 7-9% dry extract. To prepare 50% ethanolic extract, finely grinded plant material (100 gm) was charged in a percolator and treated with ethanol: water (1:1 v/v; 500 ml) at 25-30°C overnight. The marc was extracted thrice by cold percolation (1:1 v/v; 500 ml×3) and the combined percolate (1000 ml) was evaporated at 40-45°C under vacuum to obtain 7-9% dry extract. The dried 50% ethanolic stem bark extract (10 gm) was suspended in 100 ml petroleum ether, followed by stirring at 10–20 rpm at 30-40°C for 30–60 min and was subsequently left overnight at room temperature (25-30°C). Petroleum ether soluble fraction was collected after filtration. The marc (residual petroleum ether insoluble extract) was treated three more times with the petroleum ether as above, to collect the total petroleum ether soluble fraction. It was concentrated at 30-40°C and finally dried under vacuum to collect 7-9% of dried petroleum ether soluble fraction. The residual petroleum ether insoluble extract, left after extraction, was air dried for 20–30 h. It was then suspended in 100 ml of chloroform followed by stirring at 10–20 rpm at 30-40°C for 30–60 min and was left overnight at room temperature. The clear chloroform soluble fraction was collected after filtration through Whatman filter paper number 1. The residual chloroform insoluble extract was treated thrice with the chloroform as above, to collect the total chloroform soluble fraction. It was concentrated at 35-45°C and finally vacuum dried to collect 17-20% of dried chloroform soluble fraction. The residual chloroform insoluble extract was air dried for 20–30 h and treated in a similar way with n-butanol to prepare the n-butanol soluble fraction.

### Isolation of compounds

The 50% ethanolic extract (10 g) from stem bark was chromatographed on silica gel (100–200 mesh, Merck, Darmstadt, Germany) using petroleum ether, ethyl acetate and methanol as eluants. The ethyl acetate and methanol soluble fractions (95:5 to 80:20 v/v) afforded 5 compounds, which were further purified by preparative Thin Layer Chromatography (TLC) using different solvents. These compounds were identified by direct comparison with the spectroscopic data (NMR and MS) of authentic samples, procured from MP Biomedicals, Ohio, Solon, USA; Life Biochem Technologies Pvt. Ltd., New Delhi, India and Sigma Aldrich Inc., St Louis, MO, USA.

### Cells

TZM-bl cells [HeLa cell line expressing high levels of CD4, HIV-1 co-receptors CCR5 & CXCR4 with β-galactosidase and luciferase as reporter genes under HIV-1 LTR promoter; [38], HEC-1A [American Type Culture Collection, Manassas, VA, USA; an endometrial adenocarcinoma cell line; [39] and HEK-293 T cells were maintained in Dulbecco’s modified Eagle’s medium (DMEM; Sigma-Aldrich Inc.) supplemented with 10% fetal bovine serum (FBS; Biological Industries, Kibbutz beitHaemek, Israel) and an antibiotic-antimycotic cocktail [Penicillin (100 units/ml), Streptomycin (100 μg/ml) and Amphotericin B (250 ng/ml); Pen-Strep-Ampho sol, Biological Industries]. Peripheral blood lymphocytes (PBLs; obtained through IRB-approved protocol) and Caco-2 cells (American Type Culture Collection; a human epithelial colorectal adenocarcinoma cell line) were cultured in RPMI supplemented with 10% FBS and antibiotic-antimycotic cocktail as mentioned above. An immortalized cell line, Vk2/E6E7 (derived from the normal human vaginal mucosa), a generous gift from Dr. Raina Fichorova (Brigham and Women’s Hospital, Boston, MA, USA) was cultured in Keratinocyte serum-free medium (ker-sfm; Gibco- Invitrogen, Carlsbad, CA, USA) supplemented with bovine pituitary extract and epidermal growth factor [EGF; Gibco-Invitrogen; [40].

### Virus generation

HIV-1_NL4.3_ (CXCR4 tropic virus) was prepared by transfecting HEK-293 T cells with proviral plasmid DNA clone pNL4.3 (AIDS Research and Reference Reagent Program [ARRRP], Division of AIDS, National Institute of Allergy and Infectious Diseases, USA) using CaPO_4_. The medium was changed 24 h post-transfection and the supernatant was harvested after 48 h of incubation and frozen at −80°C. The concentration of virus stock was determined by the HIV-1 p24 Antigen Capture Assay ELISA (SAIC-Frederick Inc., NCI-Frederick, USA) and by determining the infectious titer using TZM-bl cells. Known inhibitors against HIV-1 infection, Azidothymidine (AZT; reverse transcriptase inhibitor; Sigma-Aldrich Inc.), Saquinavir (a protease inhibitor; ARRRP), Nevirapine (RT inhibitor; ARRRP) and Bicyclam (CXCR4 fusion inhibitor; ARRRP) were used as experimental controls.

### Anti-HIV-1 activity study

#### Cell-free virus based assay using TZM-bl cells

TZM-bl cells (4.0×10^4^/well) were seeded in 24-well plate and cultured overnight. In separate vials, HIV-1_NL4.3_ at a multiplicity of infection (MOI) of 0.05 whereas HIV-1_BaL_ at TCID_50_, were treated with extracts/fractions or solvents used to prepare above extracts/fractions for 1 h at 37°C. Subsequently, pretreated viruses were added in duplicate to TZM-bl cells and cultured for 4 h. The cells were washed once with cold 50 mM PBS, pH-7.4 to remove the cell-free virus followed by addition of fresh culture medium with extracts/fractions and further incubated for 48 h in humidified atmosphere of 5 CO_2_ at 37°C. AZT was used as a positive reference control whereas negative control comprised of cells without HIV-1 infection. After incubation, cells were washed twice with PBS and lysed with 1X lysis buffer (Promega Corporation, Madison, USA) by freeze-thaw. The supernatant was analyzed for luciferase activity by BrightGlo Luciferase Assay kit (Promega Corporation) in white opti-plate and luminescence was read using Fluorimeter (BMG Labtech GmbH, Offenberg, Germany) at a spectral range of 240–740 nm. The n-butanol fraction prepared from *A. catechu* was also evaluated for its efficacy against CCR5-tropic clinical isolates using the same protocol except that the culture medium in addition contain DEAE-dextran hydrochloride (50 μg/ml, Sigma-Aldrich Inc).

#### Cell-associated virus based assay using peripheral blood lymphocytes (PBLs)

Experiments using human blood cells were carried out under informed consent and following the clearance from the Institutional Bio-safety and Human Ethical Committee. Blood (5 ml) was taken from healthy HIV-1 seronegative donors and lymphocytes were isolated using Ficoll density gradient method. Cells (2.0×10^6^ cells/ml) were stimulated with phytohemagglutinin (PHA-P; Sigma-Aldrich Inc.) at 3 μg/ml for 3 days. After stimulation, cells were washed twice with PBS. PBLs were infected with HIV-1_NL4.3_ at an MOI of 0.05, in presence of IL-2 (10 U/ml) for 4 h. Cells were washed twice with plain medium to remove the unbound virus and seeded in 96-well plate (5.0×10^4^/well/200 μl) in RPMI medium supplemented with 10% FBS and recombinant human IL-2 (10 U/ml). Plate was further incubated at 37°C, 5% CO_2_ after adding the n-butanol fraction at varying concentrations to the wells. Cell culture supernatant was collected on 5^th^ day for p24 analysis. The culture supernatant (100 μl) was further used for infection of TZM-bl cells for 4 h, and luciferase expression was estimated after 48 h as described above to validate the infectivity of virion particles released by PBLs as an effect of treatment with n-butanol fraction of *A. catechu*.

#### p24 ELISA

Virus in the culture supernatant of n-butanol fraction/AZT treated human PBLs was quantitated by p24 estimation using an ELISA kit (SAIC-Frederick Inc., NCI-Frederick, USA; XpressBio, Life Science Products, MD, USA), following the instructions of the manufacturer. Virus load in uninfected cells was used as negative control whereas virus load in solvent treated infected cells was the virus control. Test control includes virus load in infected PBLs treated with either n-butanol fraction/AZT.

### Study of mechanism of inhibition of HIV-1 infection

#### HIV-1 entry assay

TZM-bl cells (4 × 10^4^) were pretreated with or without plant n-butanol fraction at 37°C for 1 h before being infected at an MOI of 0.05. Bicyclam was used as a reference control. The infected cells were trypsinized and washed with PBS to remove the unattached virus and cultured in fresh medium. After 48 h incubation, percent inhibition in HIV-1 infection was determined as described.

#### Env-dependent fusion assay

This bioassay allows testing for inhibitors capable of interfering with the CD4-Env interaction [41]. It employs HL2/3 cell line that express Gag, Env, Tat, Rev, and Nef, but not reverse transcriptase and secretes the env protein in the medium. HL2/3 cells (2.5 × 10^4^) were treated with the butanol fraction (25 μg/ml) in separate vials for 30 min and washed thereafter. Pre-treated HL2/3 cells were then incubated with untreated TZM-bl cells (2.5 × 10^4^) for another 30 min. Cells were then seeded in a 24-well plate (2.5 × 10^4^ of each cell type/well) and incubated at 37°C in 5% CO_2_. Fusion was readily detected microscopically after 8 h incubation. As a result of fusion, the luciferase as well as β-galactosidase gene under the LTR promoter gets expressed in the reporter TZM-bl cells. For quantitation of luciferase expression, the cells were harvested after 36 to 48 h, lysed in 100 μl of 1X lysis buffer and luciferase activity was estimated as described above.

#### Alu-HIV-1 integration PCR

To examine HIV-1 DNA integration, semi-quantitative nested Alu-LTR PCR was done as described previously [31,42]. The following primers were used for the first round of amplification:

Alu-gag (Alu Forward): 5′GCCTCCCAAAGTGCTGGGATTACAG3′

Alu-gag (gag Reverse): 5′GTTCCTGCTATGTCACTTCC-3′

The primers for second round were;

RU5 (R forward): 5′-TTAAGCCTCAATAAAGCTTGC C-3′

RU5 (U5 Reverse): 5′-GTTCGGGCGCCACTGCTAGA-3′

Genomic DNA (200 ng) prepared from TZM-bl cells infected with HIV-1_NL4.3_ in presence or absence of n-butanol fraction prepared from *A. catechu* or Raltegravir (integrase inhibitor) was used as a template for amplification with the first set of Alu-HIV-1 PCR primers in a 25 μl PCR mix. Amplification cycles were 95°C for 5 min followed by 39 cycles of 95°C for 15 sec, 50°C for 15 sec, and 72°C for 3.5 min. In the nested step, 4.0 μl of the first PCR product was used as a template in a 25 μl reaction volume and was amplified for 35 cycles using a similar PCR protocol as noted earlier. GAPDH was amplified as an internal control from 200 ng genomic DNA as a template using the cycle program as 95°C for 10 min followed by 39 cycles of denaturation at 95°C for 30 sec, 60°C for 40 sec, and 72°C for 40 sec. The amplified PCR products were resolved on 2% agarose gel and visualized by ethidium bromide staining.

#### HIV-1 reverse transcriptase (RT), protease and integrase inhibition assays

The inhibitory activity of the active n-butanol fraction of *A. catechu* on HIV-1 RT, protease and integrase was determined as per the manual’s instructions of the respective kits (RT assay kit from Roche Applied Sciences, Mannheim, Germany; Protease assay kit from Anaspec, CA, USA and Integrase Assay kit from XpressBio, Life Science Products, MD, USA; http://www.xpressbio.com/sites/default/files/EZ-1700hiv_integrase_wildtype_kit%20v%203.0_pi_061411_0.pdf).

#### Tat-inhibitor assay

TZM-bl cells (4.0×10^4^/well) were seeded in 24-well plate and cultured overnight. The cells were transfected with pTat (0.1 μg/well; ARRRP, USA) using Lipofectamine reagent (Invitrogen) according to the manufacturer’s instructions. After 4 h of transfection, cells were treated with the active plant fraction at 37°C. Forty-eight hours post-treatment, luciferase activity was measured as described above.

#### Quantitative reverse transcriptase-polymerase chain reaction (qRT-PCR)

Total RNA was isolated using the Tri reagent (Sigma Aldrich Inc.) following the standard protocol from TZM-bl cells transfected with pTat as described above. The isolated RNA (1 μg) was used to prepare the cDNA using random hexamers, dNTP mixture, RT buffer and Superscript III reverse transcriptase following the manufacturers’ protocol. The expression level of mRNA has been verified by qRT-PCR using luciferase gene specific primers. The forward and reverse primers used were 5′ ACCGCAAGTGGGGCTTCTGC 3′ and 5′ CGTGGCCAAACTCGTGGGCT 3′, respectively. The PCR parameters used were initial denaturation for 10 min at 95°C, and 40 cycles of 95°C for 15 s followed by amplification for 1 min at 62°C. Average threshold cycle (Ct) values for 18S rRNA (run in parallel reactions to the genes of interest) were used to normalize the average Ct values of the gene of interest. These values were used to calculate the average for each group from different experiments and the relative δCt was used to determine the change in the expression between the groups.

#### Tat-electrophoretic mobility shift assay (EMSA)

Tat-EMSA was performed using TAT-EMSA kit (Panomics, Inc., Redwood City, CA, USA), following user’s manual. Biotin labelled transactivation factor (TF, transactivating regulatory protein) and cold (unlabelled) TF DNA probes were provided by the manufacturer [32]. Nuclear extracts that served as a source of Tat protein were prepared from transfected TZM-bl cells. TZM-bl cells were transfected with pTat as described above. Cells were washed twice with PBS and nuclear extracts were prepared using NE-PER Nuclear and Cytoplasmic Extraction Kit (Pierce, Rockford, IL, USA). Protein concentration was determined by the BCA protein estimation kit (Pierce). Briefly, TF DNA probe was incubated with nuclear extract (4 μg) for 30 min either in the presence of n-butanol fraction (50 μg/ml) of *A. catechu* or cold TF DNA probe provided in the kit. To determine the mobility shift of the DNA probe in presence or absence of plant fraction/cold DNA probe, the samples were run in a non-denaturing polyacrylamide gel, as suggested by the manufacturer at 120 V until the dye had migrated 2/3 of the way down the gel. The gel was then transferred to a nylon membrane, UV-cross linked and biotin-labelled DNA was detected by chemiluminescence.

### Safety studies of plant extracts and n-butanol fraction

#### Cytotoxicity assessment by MTT assay

Cytotoxicity of extracts/fractions was assessed using MTT [3-(4,5-dimethylthiazol-2-yl)-2,5-diphenyltetrazolium bromide; Sigma-Aldrich Inc.] assay [43]. Briefly, TZM-bl cells (6.0 × 10^3^/well/100 μl) were seeded in a 96-well culture plate (Greiner Bio-One, GmbH, Frickenhausen, Germany) and grown overnight at 37°C in a humidified atmosphere of 5% CO_2_. Next day, culture medium with increasing concentrations of various extracts/fractions was added in duplicate and further incubated for 48 h. Appropriate solvents, used to prepare various extracts/fractions were included as negative controls. After incubation, 20 μl of MTT reagent (5 mg/ml) was added per well and incubated at 37°C for 3 h followed by addition of MTT solvent (100 μl/well; 20% SDS and 50% dimethyl formamide in 50 mM PBS). The absorbance (OD) was read at 570 nm with reference filter at 690 nm. Cell viability was calculated using the equation,

$$\begin{array}{ll} \%\;\mathrm{Viability}= & \;\left({\mathrm{OD}}_{\mathrm{Extract}/\mathrm{fraction}\;\mathrm{treated}\;\mathrm{cells}}/{\mathrm{OD}}_{\mathrm{Untreated}\;\mathrm{cells}}\right)\\ & \times 100 \end{array}$$

#### Estimation of pro-inflammatory cytokines secreted by human cervico-vaginal keratinocytes

To study, if plant extracts/fractions induce epithelial toxicity and inflammation, their impact on a human cervico-vaginal keratinocyte cell line (Vk2/E6E7) viability and analysis of pro-inflammatory cytokines secretion was done. Cervico-vaginal Vk2/E6E7 cells (6.0 × 10^3^/well) were seeded in 96-well culture plate and incubated in humidified atmosphere of 5% CO_2_ at 37°C for 24 h. After incubation, cells were treated with varying concentrations of plant extracts for 24 h and culture supernatant was collected for various cytokines quantitation using BDTM Cytometric Bead Array kit (BD FACSCanto Flow Cytometer; BD Biosciences Pharmigen, San Diego, CA, USA). The kit allowed simultaneous quantification of interleukin-10 (IL-10), IL-12 (p70), IL-1β, IL-6, IL-8 and tumor necrosis factor (TNF). The cytokine bead assay was performed according to the manufacturer’s specifications and data analysis was done using BD FACSDiva software. In addition, Vk2/E6E7 cells viability after 24 h treatment with test extracts and n-butanol fraction was also determined by MTT assay as described above.

#### Transepithelial resistance (TER) measurement

Epithelial cells monolayer integrity, lining the reproductive tract is critical for the prevention of sexual transmission of HIV-1. Transepithelial resistance (TER), a measure of epithelial integrity, was performed. Caco-2/HEC-1A cells (5.0 × 10^5^/well) were grown in appropriate medium (1 ml) in the apical chamber of transwell plates and culture medium (1.5 ml) was dispensed in the basolateral compartment of each well. The cells were allowed to grow for 36–48 h in 5% CO_2_ at 37°C and assessed for formation of monolayer by measuring TER. Resistance was measured using Millicell–ERS voltmeter (EMD Millipore Corporation, Billerica, MA, USA) each day until resistance reached plateau. After formation of monolayer, the plant n-butanol fraction (50 μg/ml) was added in the culture medium and was further incubated in humidified atmosphere of 5% CO_2_ at 37°C. Resistance was measured at 30 min, 1, 2, 4, 8 and 24 h after addition of either n-butanol fraction from *A. catechu* or nonoxynol-9.

### Calculation of percent inhibition of infection

Percent inhibition was calculated from luciferase/p24 content, utilizing the following formula:

$$\mathrm{Inhibition}\left(\%\right)\;\left[\frac{\mathrm{Virus}\;\mathrm{control}-\mathrm{Test}\;\mathrm{sample}}{\mathrm{Virus}\;\mathrm{control}-\mathrm{Mock}\;\mathrm{infected}}\right]\times 100$$

#### Statistical analysis

All studies were performed at least three times except where noted in the figures legend. Means and their standard errors are shown. Analyses of concentration-response data were performed by the use of nonlinear curve-fitting program Prism to determine CC_50_ and IC_50_ values. Student’s *t*-test was used for quantitative variables for comparison between the different groups.

## Conclusion

In conclusion, the extracts prepared from the stem bark of *A. catechu* exhibited potent anti-HIV-1 effect as demonstrated using different *in vitro* assays including human peripheral blood lymphocytes. However, the active components responsible for the activity are yet to be explored. The suppression of HIV-1 infection may be, in part due to its inhibitory effect on HIV-1 protease and partly due to the interference in interaction of viral Tat protein to the HIV-1 promoter sequence of LTR, which is also reflected by the expression of gene (luciferase) that is under the control of Tat-LTR mediated transactivation. Initial safety studies of the active n-butanol fraction from 50% ethanolic extract using vaginal keratinocytes for pro-inflammatory cytokines secretion and dual chamber model for epithelial toxicity indicates that the stem bark extract of the plant have the potential to be developed as an anti-HIV-1 microbicide candidate. Multiple mode of action of this plant has an added advantage and suggests that further safety and efficacy studies using *in vivo* models are needed. Several attempts have been made at screening numerous traditional plants as medicines in search for plant based anti-HIV-1 agents. However, this is the first report for investigation of *A. catechu* stem bark extracts for its anti-HIV-1 activity, where we showed its *in vitro* anti-HIV-1 property through the inhibition of both HIV-1 protease and LTR-Tat protein interaction. The active extracts, n-butanol fraction and catechins being less toxic confirm the specific inhibition against HIV-1 infection. In the studies done by Marquez *et al*., [44], anti-viral compounds derived from plants that interfere with HIV-1 LTR promoter regulatory proteins are not likely to generate drug-resistant HIV-1 strains, hence suggesting the important role of this plant as a traditional medicine. Further studies to identify the active constituents from *A. catechu* responsible for anti-HIV-1 activity are in progress.

## Abbreviations

HIV-1: Human immunodeficiency virus-1; HPLC: High performance liquid chromatography; LTR: Long terminal repeat; IL: Interleukin; RT: Reverse transcriptase; TLC: Thin layer chromatography; MOI: Multiplicity of infection.

## Competing interests

The authors declare that they have no competing interests.

## Authors’ contributions

SKG designed and coordinated the overall study and wrote the manuscript. SM coordinated with SK, AKS and SKS for collection and extraction of plant material. SM and SK isolated and characterized the compounds from the 50% stem bark extract of *A. catechu*. SKG, N and CSD designed and performed the anti-HIV-1 efficacy and safety experiments. MM helped in performing the experiments as well as data analysis. UR and AV prepared and titrated clinical isolates of HIV-1. All authors read and approved the final manuscript.

## Supplementary Material

Additional file 1: Figure S1 HPLC profiles of extracts from stem bark of *A. catechu.* X-axis represents time and Y-axis represents voltage*.* Solvent used was: Acetonitrile: H_2_O (18: 82 v/v; 0.5% acetic acid); at 280 nm; a) 50% Ethanolic extract; b) Aqueous extract. Click here for file

Additional file 2: Figure S2 HPLC of fractions of 50% ethanolic stem bark of *A. catechu*. X-axis represents time (in mins), whereas Y-axis represents the absorbance at 280 nm; Solvent: Acetonitrile: H_2_O (18: 82 v/v; 0.5% acetic acid); a) Petroleum ether soluble fraction; b) Chloroform soluble fraction; c) n-Butanol soluble fraction. Click here for file

Additional file 3: Figure S3 Cytotoxicity of n-butanol fraction on PBLs. The figure shows the cytotoxicity of n-butanol fraction from *A. catechu* on HIV-1_NL4.3_ infected PBLs after 5 days treatment determined by MTT assay as described in *Materials and Methods*. Y-axis shows the percent viability of cells. Click here for file

Additional file 4: Figure S4 Mechanism of inhibition by n-butanol fraction at pre-integration steps of HIV-1. A) Env-mediated cell based fusion assay. A cell-based fusion assay was used to mimic the gp120-CD4 mediated fusion of the viral and host cell membranes. HL2/3 cells were pre-incubated with n-butanol fraction from *A. catechu* (25 μg/ml) prior to incubation with untreated TZM-bl cells for fusion. Bicyclam (1 μg/ml) was used as positive reference control. B) The effect of n-butanol fraction of *A. catechu* against HIV-1 Reverse Transcriptase (RT) activity at 50 ug/ml as compared with the reference control, Nevirapine (1 μM). Y-axis represents the percent inhibition. C) The inhibitory activity of n-butanol fraction on HIV-1 integrase activity at 50 μg/ml and sodium azide (1.5%) used as positive control. Y-axis represents the percent inhibition in HIV-1 integrase activity. Values are expressed as mean ± SE of 2 different experiments performed in duplicates. Click here for file
