# Supplementary figures and images for: Extracts from Acacia catechu suppress HIV-1 replication by inhibiting the activities of the viral protease and Tat
Source: Virol J. 2013 Oct 18;10:309. doi: 10.1186/1743-422X-10-309 (PMC3819669; doi:10.1186/1743-422X-10-309)

## Slide 1
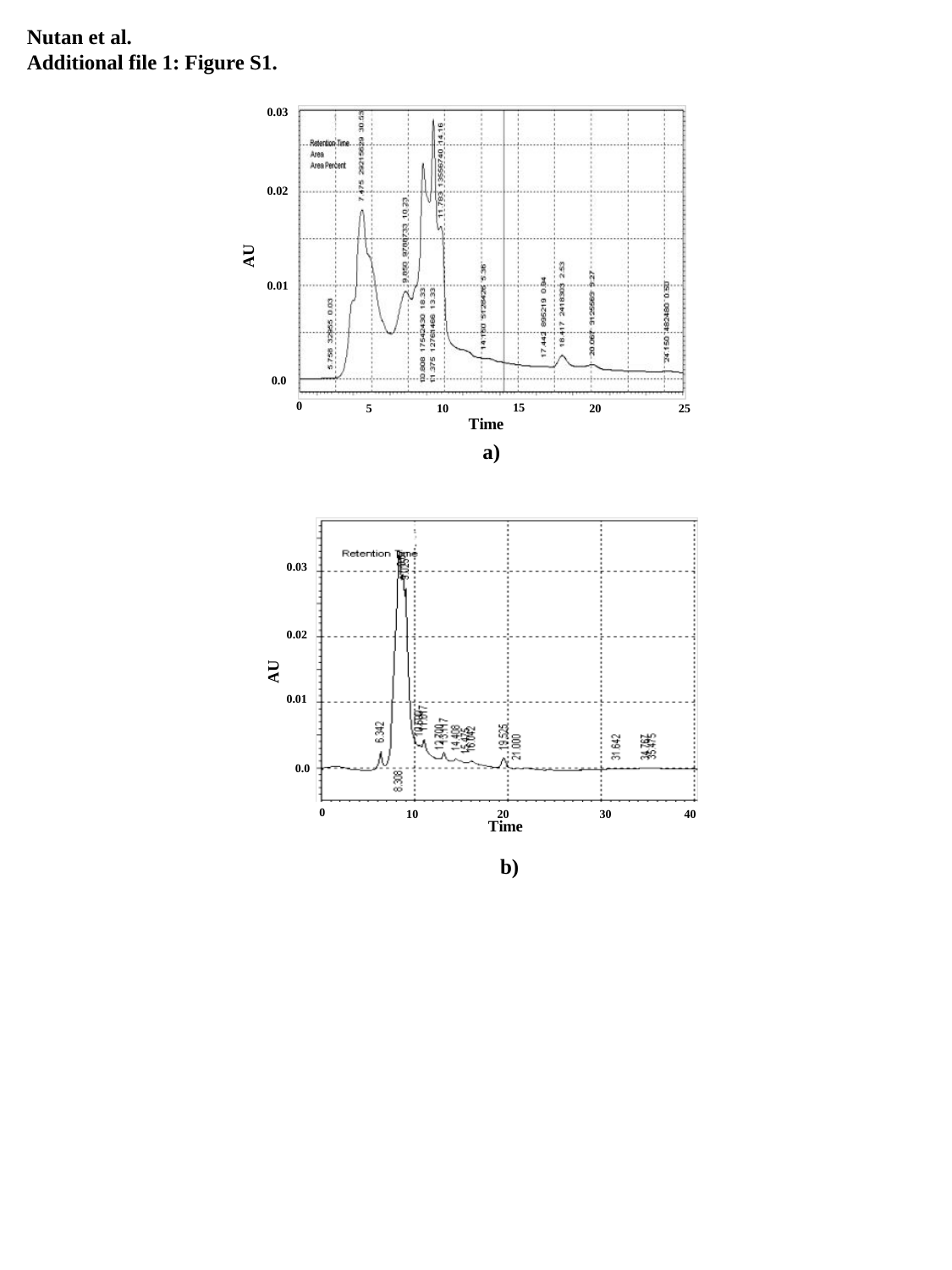

Nutan et al.
Additional file 1: Figure S1.
a)
b)

Supplement: Additional file 1: Figure S1 — HPLC profiles of extracts from stem bark of A. catechu. X-axis represents time and Y-axis represents voltage. Solvent used was: Acetonitrile: H2O (18: 82 v/v; 0.5% acetic acid); at 280 nm; a) 50% Ethanolic extract; b) Aqueous extract. [file 1743-422X-10-309-S1.pptx]

## Slide 1
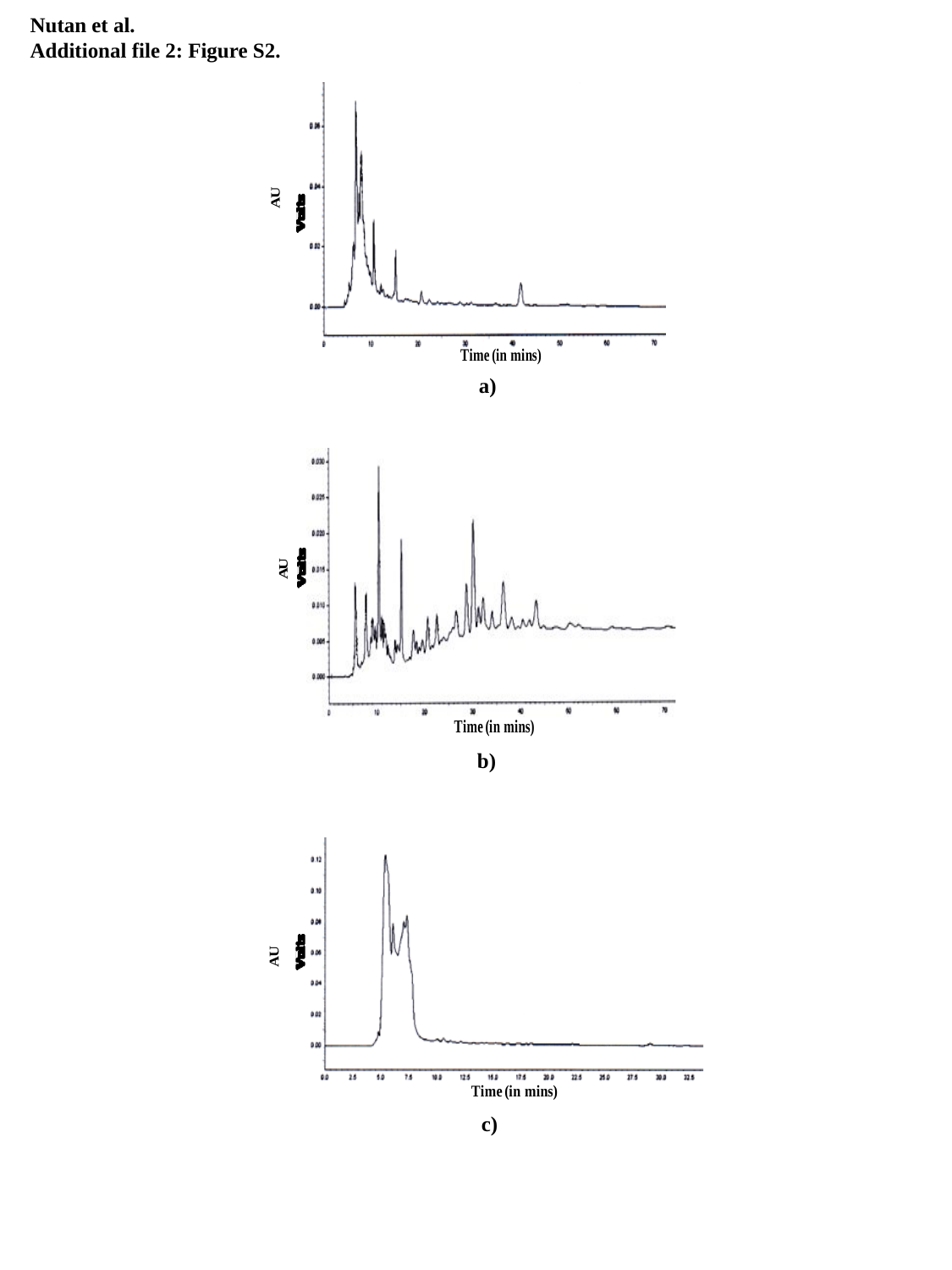

Nutan et al.
Additional file 2: Figure S2.
a)
b)
c)

Supplement: Additional file 2: Figure S2 — HPLC of fractions of 50% ethanolic stem bark of A. catechu. X-axis represents time (in mins), whereas Y-axis represents the absorbance at 280 nm; Solvent: Acetonitrile: H2O (18: 82 v/v; 0.5% acetic acid); a) Petroleum ether soluble fraction; b) Chloroform soluble fraction; c) n-Butanol soluble fraction. [file 1743-422X-10-309-S2.pptx]

## Slide 1
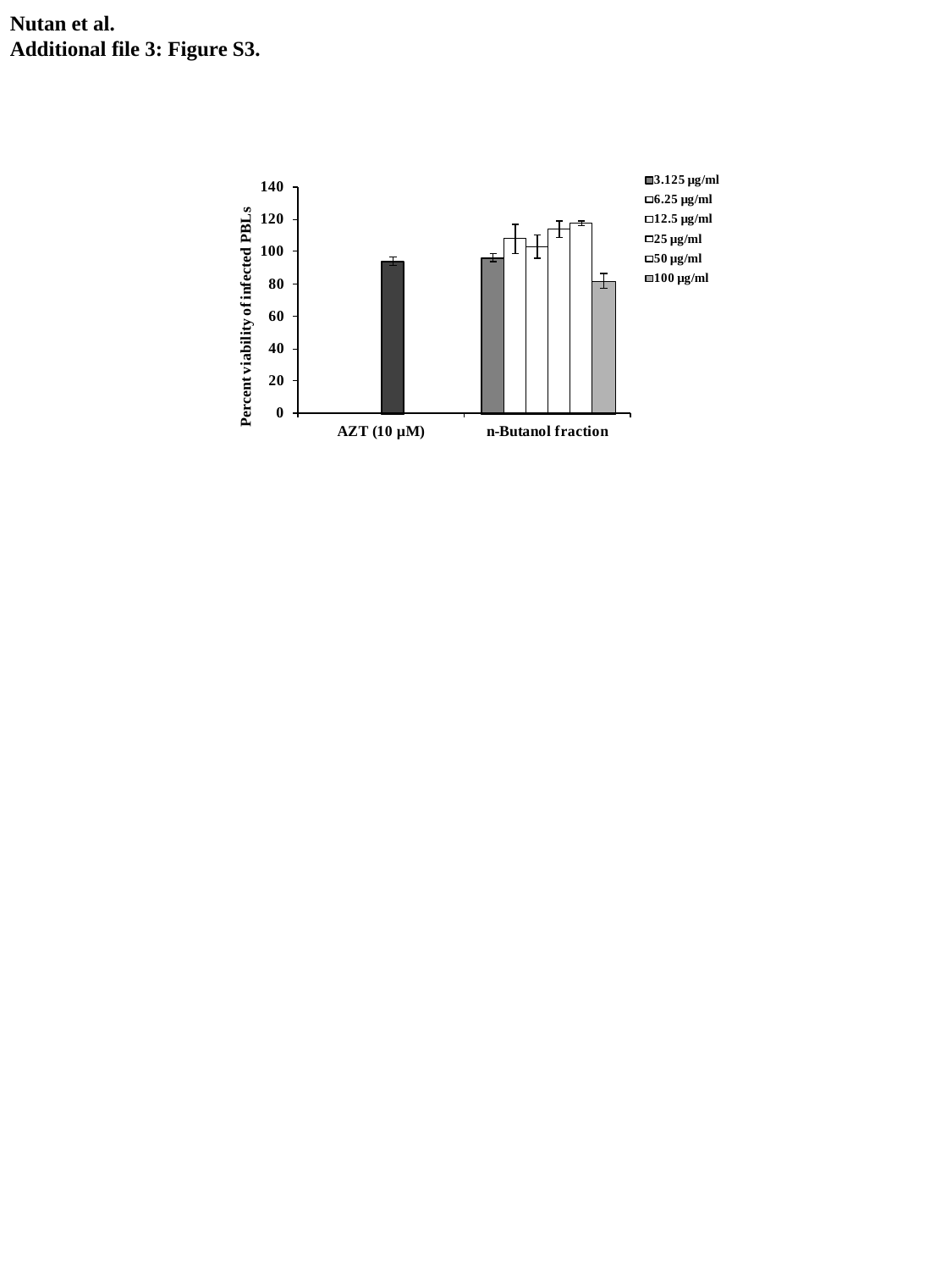

Nutan et al.
Additional file 3: Figure S3.

Supplement: Additional file 3: Figure S3 — Cytotoxicity of n-butanol fraction on PBLs. The figure shows the cytotoxicity of n-butanol fraction from A. catechu on HIV-1NL4.3 infected PBLs after 5 days treatment determined by MTT assay as described in Materials and Methods. Y-axis shows the percent viability of cells. [file 1743-422X-10-309-S3.pptx]

## Slide 1
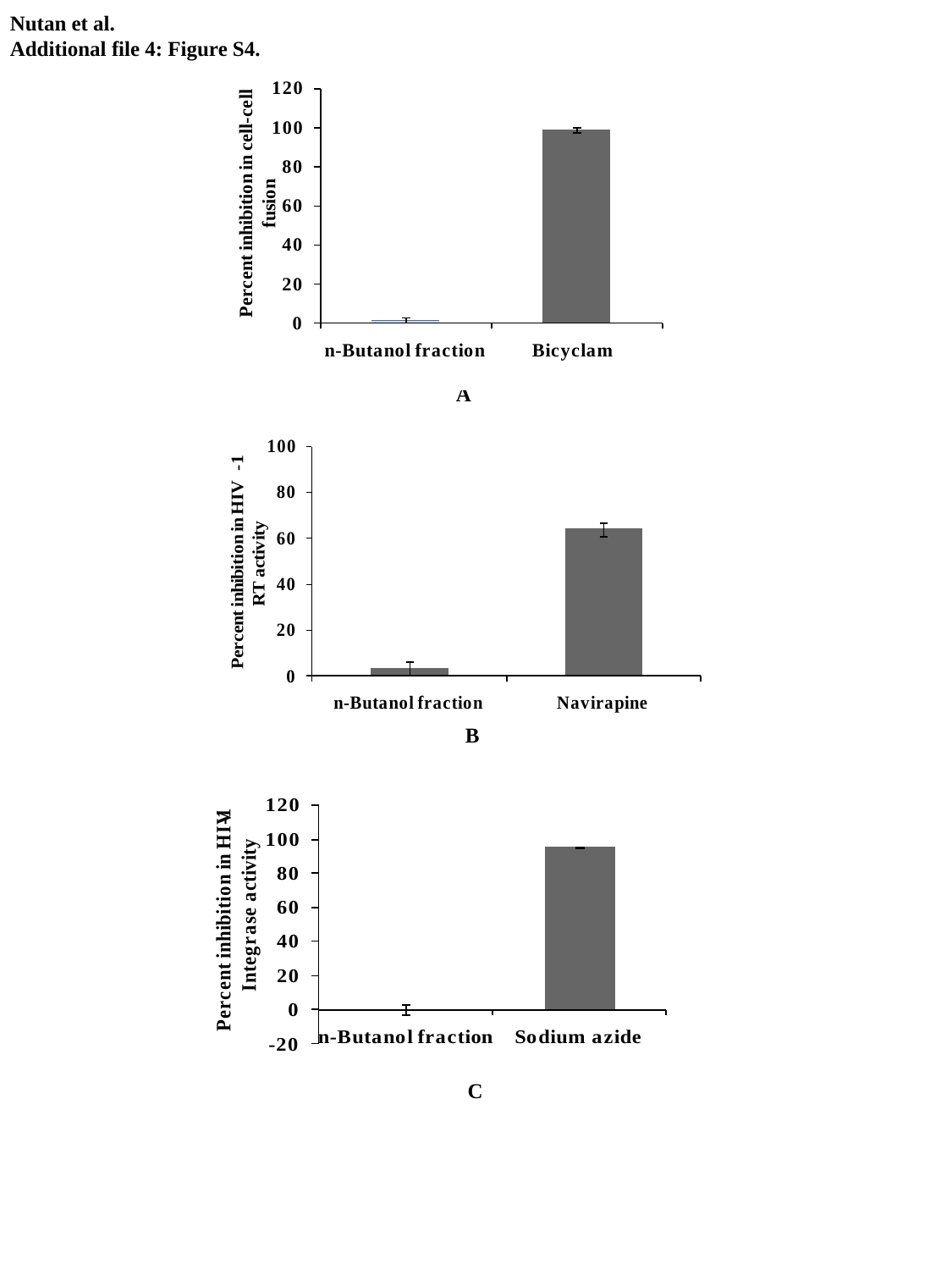

Nutan et al.
Additional file 4: Figure S4.
A
B
C

Supplement: Additional file 4: Figure S4 — Mechanism of inhibition by n-butanol fraction at pre-integration steps of HIV-1. A) Env-mediated cell based fusion assay. A cell-based fusion assay was used to mimic the gp120-CD4 mediated fusion of the viral and host cell membranes. HL2/3 cells were pre-incubated with n-butanol fraction from A. catechu (25 μg/ml) prior to incubation with untreated TZM-bl cells for fusion. Bicyclam (1 μg/ml) was used as positive reference control. B) The effect of n-butanol fraction of A. catechu against HIV-1 Reverse Transcriptase (RT) activity at 50 ug/ml as compared with the reference control, Nevirapine (1 μM). Y-axis represents the percent inhibition. C) The inhibitory activity of n-butanol fraction on HIV-1 integrase activity at 50 μg/ml and sodium azide (1.5%) used as positive control. Y-axis represents the percent inhibition in HIV-1 integrase activity. Values are expressed as mean ± SE of 2 different experiments performed in duplicates. [file 1743-422X-10-309-S4.pptx]
